# Quantitative MRI Assessment of Supraspinatus Tendon Remodeling Following a Single Platelet-Rich Plasma Injection Using *T*_2_ Mapping and Relaxation Time Profiling

**DOI:** 10.3390/diagnostics15081049

**Published:** 2025-04-21

**Authors:** Karlo Pintarić, Andrej Vovk, Vladka Salapura, Žiga Snoj, Jernej Vidmar

**Affiliations:** 1 Institute of Radiology, University Medical Centre Ljubljana, 1000 Ljubljana, Slovenia; 2 Department of Radiology, Faculty of Medicine, University of Ljubljana, 1000 Ljubljana, Slovenia; 3 Centre of Clinical Physiology, Medical Faculty, University of Ljubljana, 1000 Ljubljana, Slovenia; 4 Institute of Physiology, Faculty of Medicine, University of Ljubljana, 1000 Ljubljana, Slovenia

**Keywords:** *T*_2_ mapping, PRP, supraspinatus tendon, tendinosis, magnetic resonance imaging

## Abstract

**Background:** Quantitative magnetic resonance imaging (MRI) techniques such as *T*_2_ mapping may detect early tendon changes following biologic therapies. This study aimed to assess the structural remodeling of the supraspinatus tendon using mean *T*_2_ values and *T*_2_ distribution profiles after an ultrasound (US)-guided single platelet-rich plasma (PRP) injection. **Methods:** Twenty-six patients with symptomatic supraspinatus tendinopathy were divided into tendinosis (*n* = 9) and partial tear (*n* = 13) groups. *T*_2_ mapping and clinical evaluations (shoulder pain and disability index questionnaire (SPADI), Constant-Murley score) were conducted at baseline and 6 months post-PRP. Mean *T*_2_ values were measured in three tendon segments (lateral, middle, and medial), and *T*_2_ profiles were compared to asymptomatic controls. **Results:** Clinical outcomes showed significant improvement in both the tendinosis and partial tear groups at the 6-month follow-up. Although no significant changes were observed in the mean *T*_2_ relaxation times across tendon segments following PRP treatment, *T*_2_ distribution profiling revealed statistically significant alterations in both groups. In the tendinosis group, post-treatment *T*_2_ profiles approached those of the asymptomatic controls, suggesting structural remodeling consistent with tendon healing. **Conclusions:** *T*_2_ mapping is an effective tool for detecting tendon remodeling following PRP therapy. Structural improvements indicative of tissue healing were observed in cases of tendinosis, but not in partial tendon tears. These findings support the use of *T*_2_ mapping—particularly the *T*_2_ distribution profiling—as a quantitative biomarker for assessing treatment response to PRP.

## 1. Introduction

The pathology of the rotator cuff is one of the most frequent causes of chronic shoulder pain and disability, with the supraspinatus tendon being the most frequently affected [1]. Supraspinatus tendinopathy is primarily a degenerative condition (tendinosis) resulting from aging and overuse, often progressing to partial or complete tendon tears. An important underlying mechanism is subacromial impingement, where the rotator cuff tendons become compressed or irritated as they pass through the narrowed subacromial space [2].

There are several treatment options for supraspinatus tendinopathy depending on its severity. Conservative options with physiotherapy are widely accepted as the first-line therapy for tendinosis and small partial tears; however, most provide poor or only empirical evidence [3]. PRP, which was introduced in 1998 by Marx et al. [4] in dentistry, is an autologous concentration of platelets in a small volume of plasma, obtained from the patient’s own blood. It is rich in growth factors such as platelet-derived growth factor (PDGF), transforming growth factor-beta (TGF-β), and vascular endothelial growth factor (VEGF), which play key roles in tissue regeneration, angiogenesis, and inflammation modulation [5]. PRP injection therapy is thought to be effective in improving symptoms in early supraspinatus tendinopathy, and it is a good treatment option for patients with chronic changes in the supraspinatus tendon who do not meet criteria for surgical management or are not content with the results of conservative treatments [6].

Studies on rotator cuff tendinopathy treatment with PRP have reported variable outcomes due to different inclusion criteria, different types of PRP, and different administration techniques [7]. Only a few studies have looked into tendon changes after PRP injections. These studies evaluated tendons with standard MRI protocols [8,9,10]. The sensitivity and specificity of conventional MRI in detecting tendon remodulation and response after therapy (e.g., PRP) have proven to be variable [11,12]. This is likely due to its limited ability to detect subtle structural changes that reflect early histological and biochemical alterations. In contrast, quantitative MRI techniques—such as *T*_2_ mapping—have shown higher sensitivity to biochemical changes in various tissues, including tendons, also when using high-field MRI systems, such as 7 Tesla [13]. Therefore, *T*_2_ mapping may serve as a useful tool for detecting early tendon healing following PRP therapy [14]. To the best of our knowledge, there are currently no clinical MRI studies available evaluating supraspinatus tendon remodulation quantitatively with *T*_2_ mapping after various PRP therapies.

The aim of this study was to quantitatively evaluate supraspinatus tendon remodulation with *T*_2_ mapping after a single US-guided PRP injection. Specifically, *T*_2_ mapping was performed by measuring mean *T_2_* relaxation times as well as analyzing distribution profiles of *T*_2_ relaxation times in three equidistant segments of supraspinatus tendons.

## 2. Materials and Methods

### 2.1. Study Population and Study Design

A monocentric, prospective study was carried out with 26 patients (12 male and 14 female), aged 35–69 years (median age 49), with imaging and clinically proven supraspinatus tendinopathy (Table 1). Additionally, four asymptomatic volunteers with appropriate supraspinatus tendons were invited to participate to obtain reference values for MR quantitative analysis.

Patients were treated with single US-guided PRP injection therapy at the Clinical Institute of Radiology, University Medical Centre Ljubljana, Slovenia, from March 2019 until June 2023. The inclusion criteria for patients with supraspinatus tendinopathy were persistent pain with shoulder disability for at least 3 months after at least one cycle of physiotherapy. The exclusion criteria included patients with a complete tear or partial tear more than half of the supraspinatus tendon thickness or previous joint injuries found on MRI, rheumatoid arthritis, known malignancy, bleeding disorders, pregnancy, or use of nonsteroidal anti-inflammatory drugs 7 days before PRP treatment, as well as previous invasive treatments of the rotator cuff tendons.

**Figure 1:**
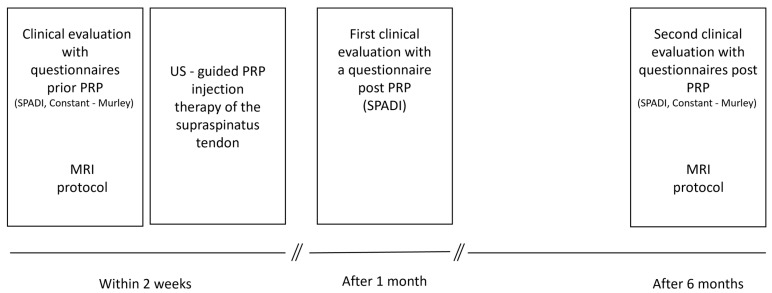
Timeline of the study protocol.

Patients were evaluated clinically prior to intervention with two standardized questionnaires: a SPADI questionnaire [15] and a Constant—Murley questionnaire [16]) and two follow-ups at 1 month with the SPADI questionnaire and after 6 months again with both questionnaires. MRI evaluation was performed prior to intervention and at 6-month follow-up. The initial clinical and MRI evaluations were 7–14 days prior to PRP injection therapy (Figure 1).

All patients were categorized into two groups: one with tendinosis of the supraspinatus tendon and the other with a small partial tear involving less than 50% of the tendon thickness. Group allocation was based on MRI criteria. Tendinosis was defined as increased signal intensity within the tendon on *T*_1_-weighted and proton-density (PD)-weighted images, consistent with degenerative changes such as collagen fiber disorganization, elevated water content, or mucoid degeneration—without signs of fiber disruption or partial tearing. Partial tears were defined as areas of hyperintense signal on *T*_2_-weighted or PD fat-saturated sequences, extending to the articular or bursal surfaces, or located entirely within the tendon substance (intratendinous) [17]. The classification was performed independently by two musculoskeletal radiologists, each with a minimum of 7 years of experience.

### 2.2. MRI Protocol

MRI examinations were performed using a 3.0 T scanner (Magnetom^®^Trio, Siemens, Erlangen, Germany) with an 8-channel transmit-receive shoulder coil (Invivo, Gainesville, FL, USA). The participants were positioned in a supine position with their palms facing upward during the examination. The imaging protocol consisted of both conventional MRI sequences and a *T*_2_ mapping sequence. Details of the imaging parameters are provided in Table 2.

### 2.3. Patient Evaluation

Two standardized questionnaires were used for the assessment of the patient’s clinical outcomes: a SPADI questionnaire and a Constant-Murley questionnaire. In the SPADI questionnaire, 0 points was assigned as the best score (i.e., no pain and no disability) and 100 points as the worst score, indicating the most severe patient’s problems [15]. In the Constant-Murley questionnaire [16], 100 points represented the best score, and 0 points was assigned to the worst score.

### 2.4. PRP Preparation

PRP preparations were obtained from autologous blood samples collected from each patient. Approximately 30 mL of blood was drawn from the untreated arm and mixed with 2 mL of anticoagulant (citrate dextrose), following the manufacturer’s instructions [18]. Centrifugation was performed using the Harvest SmartPrep 3 system (Terumo BCT, Lakewood, CO, USA), resulting in the production of 3 mL of PRP. Each patient received an injection of 2 mL of PRP. In every second patient (13 patients in total), 1 mL of the PRP was used for the analysis of platelet and leukocyte concentrations to verify consistency with the manufacturer’s specifications.

### 2.5. PRP Injection

Patients were in a supine position; surgical disinfection was performed, and a sterile probe sleeve was used. Prior to PRP application, approximately 3 mL of local anesthetic (Xylocaine^TM^) was infiltrated in subcutaneous tissue and in subacromial-subdeltoid bursae, avoiding infiltration of the tendon [19]. Then, a 2 mL PRP injection was performed under US guidance. The peppering technique was used to inject the most heterogeneous area of the tendon that corresponded to tendinosis or partial tear changes depicted on the MRI [20]. The US-guided application of PRP was performed in the lateral segment of the supraspinatus tendon close to the tendon footprint, where, in concordance with the MRI, target lesions were present. We tried to avoid the overexpansion of the tendon and retrograde leakage of the PRP along the needle outside of the tendon. All PRP applications were carried out by two radiologists subspecialized in musculoskeletal radiology with at least 7 years of practice.

### 2.6. Image Analysis

*T*_2_ maps were calculated from the eight obtained images in the coronal plane with increasing TE. Segmentation of the tendons was performed manually in the calculated coronal *T*_2_ maps comparatively to the coronal PD-TSE FS sequence, which was used simultaneously as an aid to differentiating synovial fluid within the subacromial-subdeltoid bursa and chemical shift artifacts from the tendon. The segmentation was performed by a musculoskeletal radiologist with at least 7 years of experience using the open-source program ITK-SNAP (PICSL, University of Pennsylvania, Philadelphia, PA, USA) [21]. A full-tendon mask—extending from the tendon insertion (footprint) to the musculotendinous junction—was generated from the *T*_2_ sequence with the shortest echo time (TE) and used for *T*_2_ value extraction. Additionally, pre- and post-PRP tendon masks for the same patient were computer-assisted and aligned to enable the accurate analysis of *T*_2_ changes over time.

To assess the variability in the *T*_2_ values regarding anatomical location along the tendon, the supraspinatus tendons in the *T_2_* maps were further divided into three equivalent segments (lateral, middle, and medial), starting from the footprint of the tendon and ending at the most medial part of the humeral head, as carried out in previous studies [22,23].

The mean *T*_2_ values for each of the three tendon segments were calculated by averaging the *T*_2_ relaxation times of all individual voxels within the respective segment.

**Figure 2:**
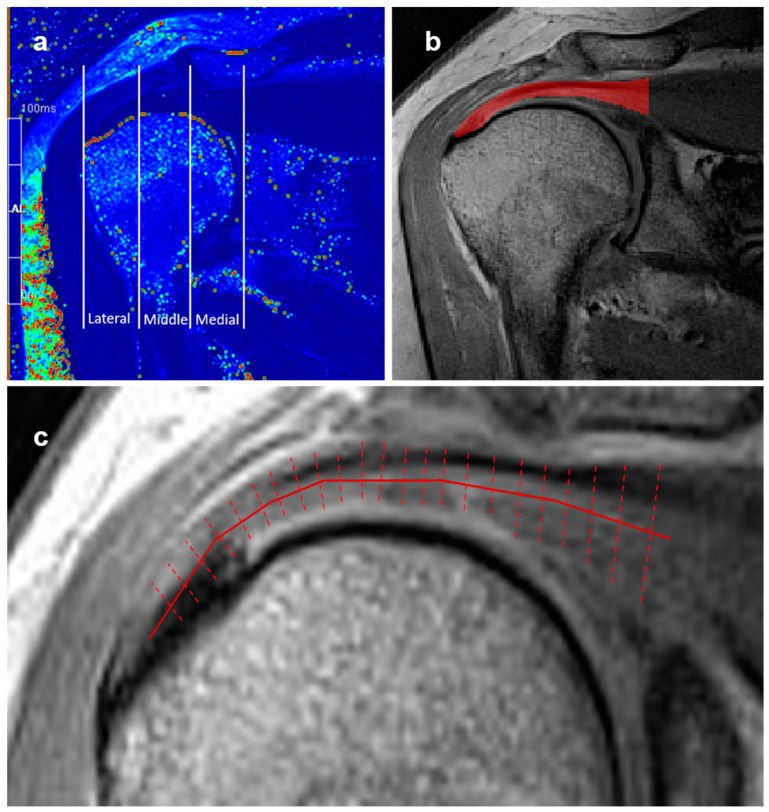
(**a**) Coronal *T*_2_ map demonstrating division of the supraspinatus tendon into three distinct anatomical segments according to the humeral head: lateral, middle, and medial segments. (**b**) Manual segmentation of supraspinatus tendon from the footprint of the tendon to the myotendinous junction (encoded in red), where *T*_2_ relaxation time analysis was performed. (**c**) Scheme of supraspinatus tendon *T*_2_ relaxation time profiling to obtain distribution of average *T*_2_ values along the tendon length.

The calculated *T*_2_ maps of tendons were processed using the R statistical software [24] to obtain *T*_2_ value distribution profiles along the tendons from the footprint to the musculotendinous junction. The procedure is illustrated in Figure 2. In order to reduce the impact of noise and the partial volume effects of bone and fluid within the subacromial-subdeltoid bursa on the *T*_2_ values of the tendons, the *T*_2_ maps were filtered first. Secondly, distributions of the *T*_2_ relaxation time profiles were obtained from the average *T*_2_ values of all voxels at a given length along the supraspinatus tendon. Finally, *T*_2_ distribution profiles were normalized with respect to the tendon length, i.e., the distance from an insertion to the musculotendinous junction. The *T*_2_ relaxation time distribution profile of asymptomatic controls with appropriate supraspinatus tendons was carried out in a similar manner to establish reference values for quantitative MRI analysis.

### 2.7. Statistical Analysis

To compare pre- and post-intervention questionnaire scores, a paired *t*-test was conducted using IBM SPSS, Version 24.0 (IBM Corp., Armonk, NY, USA) [25]. A significance level of *p* = 0.05 was used. This test was selected due to its suitability for comparing two related samples—in this case, repeated measurements (pre- and post-intervention) from the same participants. The normality of the data distributions was assessed using the Shapiro–Wilk test.

To evaluate changes in the mean *T*_2_ relaxation times within distinct tendon segments before and after PRP treatment in both the tendinosis and partial tear groups, the nonparametric Wilcoxon signed-rank test was used.

To investigate temporal changes in the *T*_2_ relaxation times within distinct tendon segments, linear regression analyses were performed independently for each group (tendinosis, partial tear, and asymptomatic controls) across three defined regions of the supraspinatus tendon. This approach allowed for the modeling of *T*_2_ trends through the tendon length within each condition and region, providing a continuous and interpretable measure of change. These analyses were conducted using the R (v4.1.2; R Core Team 2021) statistical environment [24]. Subsequently, the Chow test within the R framework was employed to statistically compare the regression parameters (slope and intercept) between the different groups within each tendon segment, allowing for the identification of significant differences in the rate of *T*_2_ value change over time. With these methods in place, we proceeded to evaluate clinical outcomes and tendon structural changes in both tendinosis and partial tear groups, as described in the following results.

## 3. Results

All patients were clinically evaluated at all time points. However, 22 (84%) patients had complete MRI evaluations, as 4 patients refused to do a follow-up MRI.

The supraspinatus tendinosis group consisted of 9 patients (8 females, median age of 50 years) and the supraspinatus partial tear group consisted of 13 patients (4 females, median age of 47 years).

Laboratory analysis of PRP samples showed that the average platelet concentration in the applied PRP was 1954 × 10^3^ platelets/μL (range 1524 × 10^3^/μL–2163 × 10^3^/μL) and the average leukocyte concentration was 24.0 × 10^3^/μL (range 15.6 × 10^3^/μL–31.5 × 10^3^/μL) with some inclusion of neutrophils.

### 3.1. Clinical Evaluation

Overall, 22 out of 26 enrolled patients stated at least some improvement of symptoms after PRP injection therapy, according to results of clinical questionnaires. There was a significant improvement in SPADI 1 month after PRP injection therapy, and the outcome improved even more 6 months after PRP injection therapy. Also, the Constant-Murley score showed significant improvement 6 months after PRP injection therapy (Table 3).

When comparing each studied group (i.e., tendinosis or partial tear) separately, statistically significant differences were found in the SPADI score after 1 month in the tendinosis group, but there were no differences in the partial tear group. However, a statistically significant improvement was observed in both questionnaires 6 months after the treatment in the tendinosis group, as well as in the partial tear group (Table 3).

### 3.2. Adverse Events After PRP Injection Therapy

Following PRP injection therapy, five patients (19%) experienced a temporary worsening of their initial symptoms. Two of these patients reported mildly reduced strength and a moderately limited range of motion in the treated arm, which persisted for up to three months before gradually resolving without further intervention. These two patients were treated with a 7-day course of non-steroidal anti-inflammatory drugs (NSAIDs) during the first week after the injection. Additionally, three patients experienced localized pain in the treated arm lasting 2–3 weeks, which subsided after a few days of paracetamol therapy. Despite these adverse events, all five patients showed at least some clinical improvement at the 6-month follow-up compared to their baseline condition.

### 3.3. Analysis of T_2_ Maps

**Figure 3:**
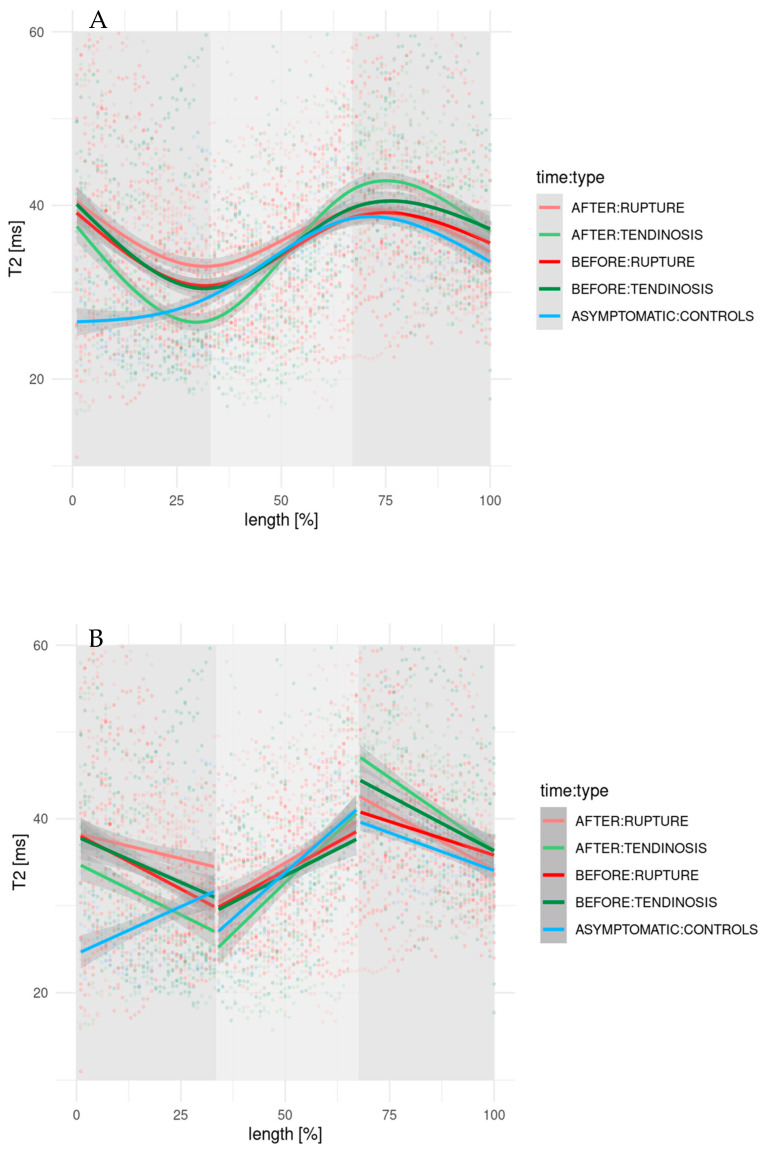
(**A**) *T*_2_ relaxation time distribution profiles, plotted by locally weighted smoothing in R and normalized with respect to the tendon length for tendinosis and partial tear group prior to and after PRP treatment. *T*_2_ distribution profiles of asymptomatic controls are shown as a reference. (**B**) *T*_2_ distribution profiles as linear regression divided into three distinct segments.

The *T*_2_ distribution profiles of supraspinatus tendons in all study groups exhibited a biphasic pattern, with distinct mean *T*_2_ relaxation times observed across the lateral, middle, and medial segments (Figure 3). The corresponding linear regression parameters (slope and intercept) for each tendon segment, both before and after PRP treatment, are presented in Table 4.

**Figure 4:**
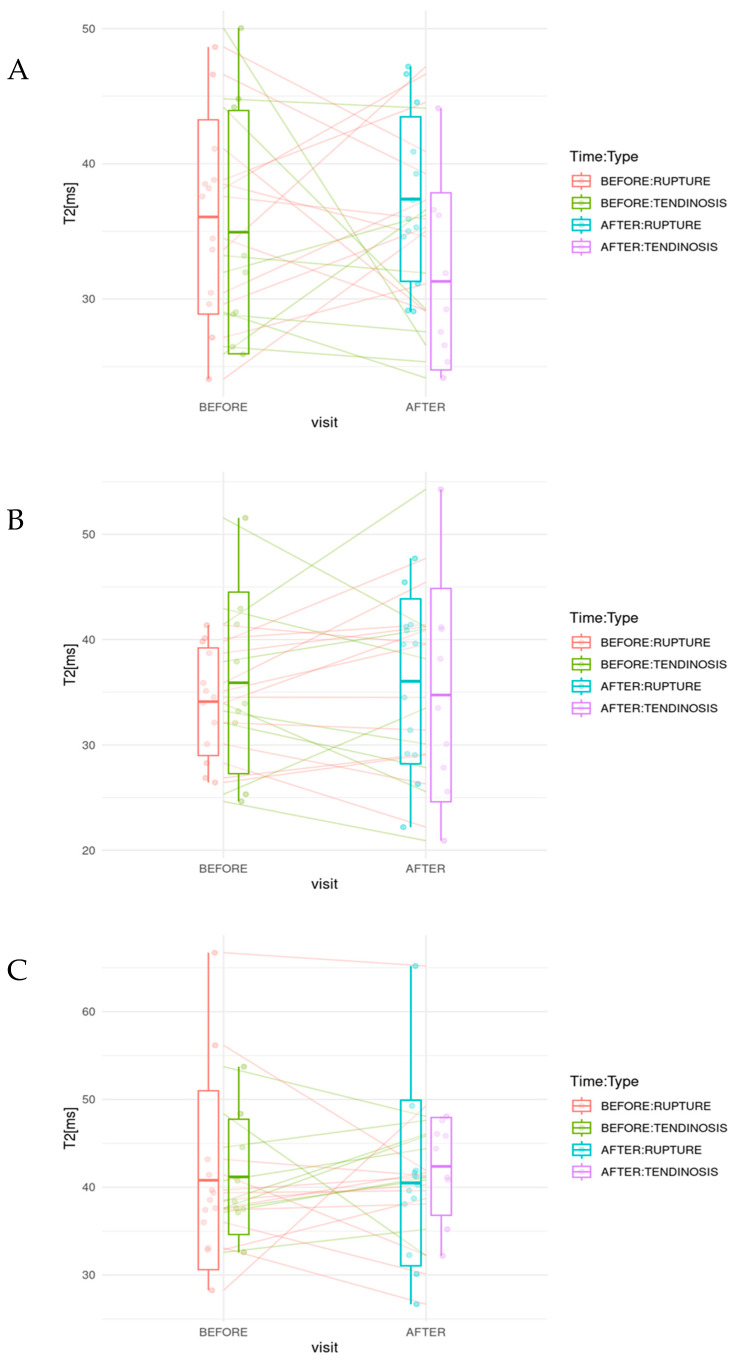
Boxplots of mean *T*_2_ relaxation times across three supraspinatus tendon segments before and after PRP treatment: (**A**) lateral, (**B**) middle, and (**C**) medial segment.

In the tendinosis group, the pre-treatment *T*_2_ profile showed a descending trend in the lateral segment, an ascending trend in the middle segment, and a descending trend in the medial segment. Following PRP therapy, this profile became less steep in the lateral segment, more pronounced in the middle segment, and remained relatively unchanged in the medial segment. Post treatment, the mean *T*_2_ relaxation times decreased in the lateral and middle segments, whereas a slight increase was noted in the medial segment (Table 5). The corresponding box plots are presented in Figure 4.

In the partial tear group, the pre-treatment *T*_2_ profile followed a similar biphasic pattern: descending in the lateral segment, ascending in the middle, and descending again in the medial segment. After PRP treatment, the lateral segment showed a less steep decline, the middle segment demonstrated a more pronounced increase, and the medial segment showed an even steeper decline. A slight increase in mean *T*_2_ relaxation times was observed in the lateral and middle segments, while values in the medial segment remained largely unchanged from the baseline.

By contrast, the asymptomatic control group exhibited a more monophasic distribution, with *T*_2_ values increasing in the lateral and middle segments and decreasing in the medial segment (Figure 3).

No statistically significant differences were found between pre- and post-treatment mean *T*_2_ relaxation times within any segment for either the tendinosis or partial tear groups (Table 5). However, significant changes were detected in *T*_2_ distribution profiles, specifically in the slopes and intercepts of the regression lines in both the lateral and middle segments of the tendinosis and partial tear groups after treatment (Table 4).

The observed trends in clinical scores and *T*_2_ values form the basis for further interpretation of the PRP treatment’s impact on tendon structure and function; these are discussed in detail in the following section.

## 4. Discussion

In the present study, analysis of the mean *T*_2_ relaxation time in three segments of the supraspinatus tendon and *T*_2_ distribution profiles along the supraspinatus tendon was utilized for the quantitative assessment of tendon response following a single PRP injection therapy. Clinical evaluation revealed a statistically significant improvement in patient conditions. However, structural changes in the tendons, characterized by decreased *T*_2_ relaxation times indicative of tendon remodeling comparable to asymptomatic controls, were observed only in patients with tendinosis.

Laboratory analysis of PRP samples showed that the average platelet concentration in the applied PRP was high, and the average leukocyte concentration was also elevated, with some inclusion of neutrophils. These findings were consistent with the concentrations advertised by the manufacturer [18]. According to the PAW classification system, the injected leukocyte-rich PRP (LR-PRP) was classified as P4-Aα [26].

In our study, most patients experienced symptomatic relief as early as one month following PRP treatment, with continued progressive improvement observed over the six-month follow-up period. However, a small subset of patients with partial tendon tears reported no clinical benefit. These results support the effectiveness of PRP therapy in treating tendinosis and small partial tears, aligning with previous findings [27,28,29]. Retrospective measurements of the tear width were conducted for all patients with partial tears, revealing no significant difference between those who responded to treatment and those who did not. The mean tear width was 8 mm in both the responsive and non-responsive subgroups.

The adverse effects observed in our study were mild, self-limiting, and consistent with previously published reports. Approximately one-fifth of patients experienced a temporary worsening of symptoms following PRP injection, including transient localized pain, mild reduction in strength, and limited range of motion—most of which resolved within a few weeks with short courses of low doses of standard analgesics. Importantly, all affected patients showed at least some clinical improvement at the 6-month follow-up. These findings align with those reported by Prodromos et al. [30], who observed only minor post-injection soreness and no major complications over a two-year follow-up, as well as by Abd Karim et al. [31], whose randomized trial reported similar safety profiles between PRP and prolotherapy groups, with only transient injection-site discomfort. Taken together, the data support the overall safety of PRP therapy for supraspinatus tendinopathy and partial tears, particularly when administered under ultrasound guidance.

Our findings agree with previous studies that have demonstrated variability in the *T*_2_ relaxation times in healthy supraspinatus tendons. We observed a similar progressive increase in the mean *T*_2_ values from the lateral to the medial side in healthy tendons of the asymptomatic controls. This trend is likely due to the gradual increase in water content near the myotendinous junction of the supraspinatus muscle, as previously reported [22]. Similarly, a progressive increase in the mean *T*_2_ values was also noted in the altered tendons prior to PRP injection therapy. However, the mean *T*_2_ values in each segment of the affected tendons remained consistently higher than those in healthy tendons, a finding consistent with previous studies [22,23,32]. The most likely explanation for these elevated *T*_2_ relaxation times is the presence of structural alterations—such as fiber disorganization associated with tendinosis and fiber disruption characteristics of partial-thickness tears. These changes are often accompanied by increased concentrations of mucoid material and interstitial water within the tendon matrix, both of which contribute to prolonged *T*_2_ values. Additionally, elevated *T*_2_ times may reflect increased vascularity and infiltration of inflammatory cells, particularly during the early stages of tendon healing or in response to acute injury.

When comparing the mean *T*_2_ values in the lateral segment across all three groups (asymptomatic controls, tendinosis, and partial tears) between our study and that of Ece et al. [32], it becomes evident that pre-treatment mean *T*_2_ values were higher in the asymptomatic and tendinosis groups in our study, whereas the partial tear group showed higher mean *T*_2_ values in their study. Several factors may explain these differences. Notably, Ece et al. [32] used a 1.5 T MRI scanner, while our study utilized a 3.0 T system, which likely provided greater signal sensitivity and may have contributed to higher *T*_2_ measurements in the asymptomatic and tendinosis groups. Additionally, their rupture group included both partial- and full-thickness tears, which likely resulted in higher *T*_2_ values due to more extensive tendon damage. In contrast, our study focused exclusively on small partial tears, associated with less structural disruption and, therefore, lower *T*_2_ elevations. Furthermore, Ece et al. [32] employed region-of-interest (ROI)-based measurements to determine mean *T*_2_ values, whereas we calculated mean *T*_2_ values using all voxels within each defined tendon segment, providing a more comprehensive analysis.

Previous studies have primarily utilized region-of-interest (ROI)-based *T*_2_ measurements, which provide a simple and efficient means of quantifying tendon pathology. However, this method may lack the sensitivity to detect subtle, regional variations within the supraspinatus tendon. In our study, although the division of the tendon into three anatomical segments and the calculation of mean *T*_2_ values from all voxels within each segment offered a more standardized approach, it may have inadvertently obscured or masked localized structural changes. This limitation could account for the minor, non-significant differences observed in mean *T*_2_ relaxation times before and after PRP therapy. To overcome this, we employed *T*_2_ distribution profiling, which involves calculating the average *T*_2_ values of all voxels at each point along the entire length of the tendon. This technique enables a more detailed and spatially refined assessment of *T*_2_ relaxation times. In our analysis, *T*_2_ distribution profiling proved to be a highly sensitive and effective method for detecting the localized structural remodeling of the tendon following PRP treatment.

The *T*_2_ distribution profile of tendons in the asymptomatic controls, used as a reference, exhibited a progressive increase in *T*_2_ values across the lateral and middle segments—consistent with the pattern observed in the analysis of mean *T*_2_ relaxation times. In contrast, prior to PRP therapy, the *T*_2_ distribution profiles in both the tendinosis and partial tear groups appeared more biphasic, with a higher overall offset in *T*_2_ values and, notably, a descending slope in the lateral segment—opposite to the trend seen in healthy tendons. This deviation is likely attributable to the most pronounced tissue alterations occurring near the tendon insertion site, where tendinosis or partial tears were typically located.

At the 6-month follow-up, *T*_2_ map analysis in the tendinosis group revealed a slight decrease in mean *T*_2_ relaxation times in the lateral and middle segments, approaching the reference values observed in the asymptomatic controls. Furthermore, post-treatment *T*_2_ distribution profiles demonstrated a significant change (i.e., a steeper slope and a reduction in offset values) in the treated lateral segment compared to pre-treatment profiles. These changes in *T*_2_ relaxation dynamics are likely indicative of positive structural remodeling induced by PRP therapy, characterized by improved longitudinal fiber organization and reduced concentrations of mucoid material and interstitial water—thus restoring tissue properties to resemble those of healthy tendons. Importantly, the observed decreases in *T*_2_ values may also reflect the resolution of local inflammation and the normalization of tissue hydration, in addition to collagen realignment and reduced matrix degeneration. The positive tissue response extended along the entire length of the lateral segment, as demonstrated by *T*_2_ distribution profile analysis, and was also observed to some extent in the middle segment, as indicated by the mean *T*_2_ relaxation times. This suggests that PRP influences tendon healing even beyond the immediate injection site [33].

In the partial tear group, an increase in mean *T*_2_ relaxation times was observed at the 6-month follow-up—somewhat unexpectedly—affecting both the lateral and middle segments of the tendon. The post-treatment *T*_2_ distribution profile exhibited a flatter slope with minimal changes in offset values. This may indicate a reduced tissue response of partial tendon tears to PRP therapy. One possible explanation for the increased *T*_2_ relaxation time could be the presence of residual fluid either from the injected PRP or fluid retained within the persistent tendon defect. Moreover, the biological response in partial tears may be more limited or delayed compared to tendinosis. Variability in tear morphology—since different types of partial tears were included in the study—could also influence the healing potential. Additionally, although a standardized US-guided injection protocol was applied, variations in PRP diffusion or retention within partial tears may have influenced outcomes. Finally, the 6-month follow-up period may have been too short to capture the full extent of tissue remodeling induced by PRP therapy in the partial tear group. Despite the absence of measurable improvements in the supraspinatus tendon structure, most patients in the partial tear group still reported subjective clinical improvement. This suggests that the beneficial effects of PRP therapy may not solely arise from tendon remodeling but may also involve pain modulation mechanisms. Laboratory analysis of PRP residues confirmed the use of leukocyte-rich PRP, which releases pro-inflammatory cytokines that could contribute to the alleviation of clinical symptoms, as was shown in previous studies [34,35].

This study has several limitations. First, although *T*_2_ mapping is a promising tool for quantitatively assessing tendon structure and monitoring tissue remodeling, its integration into routine clinical practice remains challenging. It requires access to specialized MRI sequences and advanced post-processing software, which may not be available in all radiology departments. Moreover, the technique is time-consuming, and the standardization of imaging protocols and segmentation methods across institutions is essential to ensure the reproducibility and comparability of *T*_2_ values.

Second, the sample size was relatively small, as this was designed as a pilot study. As a result, greater variability in measurements and less robust linear regression fits were observed. Despite this, the study successfully demonstrated the feasibility of using *T*_2_ mapping to capture tendon response to PRP therapy in both tendinosis and partial tear cases. A priori calculations based on expected clinical outcomes indicated that at least eight participants would be sufficient to detect meaningful effects. However, no formal power analysis was conducted for imaging endpoints (e.g., mean *T*_2_ relaxation times) due to the exploratory nature of the study. A post hoc power analysis confirmed that the available sample size provided adequate power to detect differences between healthy and pathological tendons. Nonetheless, subgroup analyses (e.g., tendinosis vs. partial tear) may be underpowered, and future studies with larger cohorts are needed to validate these findings.

Third, the study did not differentiate between specific types of partial-thickness tears—namely, bursal-sided, articular-sided, or intratendinous tears. Although inclusion criteria were limited to partial tears involving less than 50% of tendon thickness, the tear width was not predefined. Retrospective measurements showed that all included tears were small, averaging less than one-third of the tendon width (mean 8 mm). Future investigations should consider stratifying tear morphology to explore its potential influence on treatment response.

A key strength of this study is the application of quantitative *T*_2_ mapping—specifically, the measurement of mean *T*_2_ relaxation times alongside *T*_2_ distribution profiles—across multiple tendon regions. This approach allows for a more comprehensive and spatially detailed analysis of *T*_2_ relaxation times along the tendon’s length. With further refinement, *T*_2_ mapping combined with *T*_2_ distribution profiling holds promise as a potential imaging biomarker for detecting structural changes during tendon healing following PRP therapy. Future studies, ideally multicenter and involving larger cohorts with extended follow-up periods, are warranted to validate these findings and to further assess their clinical relevance.

In summary, the results of this study highlight the potential of quantitative MRI—particularly *T*_2_ distribution profiling—to be used as a sensitive and informative tool for evaluating tendon responses to biologic therapies. The key conclusions are summarized below.

## 5. Conclusions

A single US-guided PRP injection represents a promising treatment option for patients with supraspinatus tendinosis or small partial tears who do not respond to conservative therapy. *T*_2_ mapping facilitates the quantitative evaluation of the tendon structure before and after PRP treatment through the segmental analysis of mean *T*_2_ relaxation times and *T*_2_ distribution profiles. Both analytical approaches revealed structural changes in tendons across both study groups following PRP therapy, with *T*_2_ distribution profiling proving particularly informative. However, tendon remodeling that resulted in characteristics resembling those of asymptomatic controls was observed exclusively in the tendinosis group.

## Figures and Tables

**Table 1:** Demographic characteristics of the study population. The study included 26 patients diagnosed with supraspinatus tendinopathy, who were further classified into two groups based on MRI findings: tendinosis (*n* = 9) and partial tear (*n* = 13). Four patients did not undergo follow-up MRI, resulting in twenty-two complete MRI datasets. Additionally, four asymptomatic volunteers were included as controls to establish baseline *T*_2_ mapping values.

Group	Number of Patients	Male (*n*)	Female (*n*)	Median Age (Years)	Age Range (Years)
Total patients	26	12	14	49	34 (35–69)
Tendinosis	9	1	8	50	21 (35–56)
Partial tear	13	9	4	47	32 (37–69)
Asymptomatic controls	4	2	2	32	2 (31–33)

**Table 2:** Magnetic resonance imaging parameters.

			Sequence			
Parameter	Sagittal PD Fat Sat	Coronal PD Fat Sat	Axial PD Fat Sat	Coronal T1	Sagittal *T*_2_	Coronal *T*_2_ Map
Repetition time (TR) (ms)	3570	3020	3370	785	6310	2000
Echo time (TE) (ms)	44	35	35	12	94	11.4 22.8 34.2 45.6 57.0 68.4 79.8 91.2
Flip angle (°)	120	120	120	120	120	180
Field of view (mm^2^)	150	150	150	150	150	140
Slice Thickness (mm^2^)	2.5	2.5	2.5	2	2.5	2
Matrix	207 × 320	256 × 320	240 × 320	250 × 320	250 × 320	256 × 256

**Table 3:** Comparison of clinical evaluation scores before and after platelet-rich plasma (PRP) injection therapy. Values are presented as means ± standard deviations, median, and interquartile ranges. The *p*-values represent results from paired *t*-tests comparing pre- and post-treatment scores of mean values. All 26 patients included in the study were clinically evaluated at all points of time.

			BEFORE			AFTER		
Questionnaire	Group	Mean	Median	Q1–Q3 Range	Mean	Median	Q1–Q3 Range	*p*-Value
SPADI (1-month after PRP)	All (*n* = 26)	52.0 ± 19.9	54.0	41.0–66.0	39.5 ± 25.1	35.0	17.0–59.0	0.007
	Tendinosis (*n* = 13)	55.0 ± 19.3	56.0	40.0–72.0	39.6 ± 24.8	31.0	16.5–60.5	0.003
	Partial tear (*n* = 13)	48.9 ± 20.8	47.0	35.5–64.0	39.3 ± 26.3	35.0	16.5–60.0	0.229
SPADI (6-months after PRP)	All (*n* = 26)	52.0 ± 19.9	54.0	41.0–66.0	17.0 ± 20.6	9.5	2.0–19.5	<0.001
	Tendinosis (*n* = 13)	55.0 ± 19.3	56.0	40.0–72.0	14.7 ± 21.9	8.0	1.0–16.5	<0.001
	Partial tear (*n* = 13)	48.9 ± 20.8	47.0	35.5–64.0	19.3 ± 19.8	14.0	4.0–32.0	0.005
CONSTANT-MURLEY (6-months after PRP)	All (*n* = 26)	40.1 ± 14.9	38.5	29.0–51.5	71.0 ± 22.0	73.0	59.5–89.0	<0.001
	Tendinosis (*n* = 13)	40.8 ± 12.5	39.0	30.5–54.0	76.5 ± 19.8	78.0	63.0–96.5	<0.001
	Partial tear (*n* = 13)	39.6 ± 17.6	38.0	27.0–50.0	67.0 ± 23.8	69.0	41.0–88.0	0.008

**Table 4:** Linear regression parameters (slope and intercept) of *T*_2_ relaxation time profiles before and after PRP treatment across three anatomical segments of the supraspinatus tendon.

		BEFORE		AFTER		
		Slope	Intercept	Slope	Intercept	*p*-Value
TENDINOSIS (*n* = 9)	Lateral	−0.268	39.5	−0.195	34.6	0.0056
	Middle	0.151	28.4	0.572	6.15	0.0014
	Medial	−0.347	70.1	−0.359	72.4	0.1849
PARTIAL TEAR (*n* = 13)	Lateral	−0.458	43.8	−0.187	40.6	0.0021
	Middle	0.264	20.9	0.402	16.0	0.0078
	Medial	−0.135	52.0	−0.284	64.2	0.1285
ASYMPTOMATIC CONTROLS (*n* = 4)	Lateral	0.217	24.5			
	Midlle	0.430	12.4			
	Medial	−0.174	51.4			

**Table 5:** Comparison of mean *T_2_* relaxation times before and after PRP between segments for tendinosis group and partial tear group. Mean *T_2_* relaxation times in all three segments of supraspinatus tendon of asymptomatic controls.

Group	Segment	BEFORE (Mean *T*_2_ [ms] ± SD)	AFTER (Mean *T*_2_ [ms] ± SD)	*p*-Value
TENDINOSIS (*n* = 9)	Lateral	34.9 ± 11.6	31.3 ± 8.9	0.2500
	Middle	35.9 ± 11.8	34.7 ± 13.5	0.4961
	Medial	41.2 ± 9.5	42.4 ± 7.9	0.4258
PARTIAL TEAR (*n* = 13)	Lateral	36.1 ± 13.0	37.4 ± 11.9	0.5417
	Middle	34.1 ± 7.6	36.0 ± 11.1	0.1677
	Medial	40.8 ± 11.6	40.5 ± 10.8	0.6848
ASYMPTOMATIC CONTROLS (*n* = 4)	Lateral	28.1 ± 6.1		
	Middle	33.9 ± 6.4		
	Medial	36.9 ± 3.3		

## Data Availability

The data that support the findings of this study are available from the corresponding authors upon reasonable request.
